# Coronary complications in Kawasaki disease: giant aneurysms and thrombosis leading to myocardial infarction

**DOI:** 10.1093/ehjimp/qyae116

**Published:** 2025-01-17

**Authors:** Jacobo Sebastián Vera-Chávez, Carlos Antonio Villegas-Chávez, Gabriela Meléndez-Ramírez, María del Carmen López-Rodríguez, Karina Del Valle Zamora

**Affiliations:** Eighth Floor Hospitalization, National Institute of Cardiology: Instituto Nacional de Cardiologia, Juan Badiano 1, Belisario Domínguez Secc 16, Tlalpan, Mexico City, Mexico 14080, Mexico; Eighth Floor Hospitalization, National Institute of Cardiology: Instituto Nacional de Cardiologia, Juan Badiano 1, Belisario Domínguez Secc 16, Tlalpan, Mexico City, Mexico 14080, Mexico; Department of Magnetic Resonance Imaging, Juan Badiano 1, Belisario Domínguez Secc 16, Tlalpan, 14080 Mexico City, CDMX, Mexico; Eighth Floor Hospitalization, National Institute of Cardiology: Instituto Nacional de Cardiologia, Juan Badiano 1, Belisario Domínguez Secc 16, Tlalpan, Mexico City, Mexico 14080, Mexico; Eighth Floor Hospitalization, National Institute of Cardiology: Instituto Nacional de Cardiologia, Juan Badiano 1, Belisario Domínguez Secc 16, Tlalpan, Mexico City, Mexico 14080, Mexico

**Keywords:** Kawasaki disease, giant aneurysm, myocardial infarction, coronary artery bypass grafting

A 28-year-old female with a prior diagnosis of Kawasaki disease at age 3, who did not receive immunoglobulin treatment, presented with a non-ST elevation myocardial infarction. She visited the emergency department due to retrosternal, transfictive chest pain radiating to both arms and back. The physical examination revealed no additional symptoms.

The patient had a cardiovascular history of myocardial revascularization surgery, including coronary artery bypass grafting (CABG) to the RCA and LCA using the mammary artery. Initially, a non-ST elevation myocardial infarction was identified (*Figure 1B*). She underwent catheterization (*Figure 1C–F*, Supplementary data online, *Videos S1* and *S2*) as part of the infarction protocol, although she refused coronary reperfusion management. Further studies, including a CT angiography, revealed a giant aneurysm in the LCA 18 × 10 mm (*Figure 1C*, *G*, and *K*), and thrombosis of the RCA aneurysm, previously measured 34 × 14 mm, thrombosed (*Figure 1F*, *H*, and *M*). Magnetic Resonance Imaging showed a transmural infarction in the inferoseptal and inferolateral regions (*Figure 1L*). An echocardiogram indicated an LVEF of 62% with mild inferior akinesia (*Figure 1A*).

**Figure 1.**
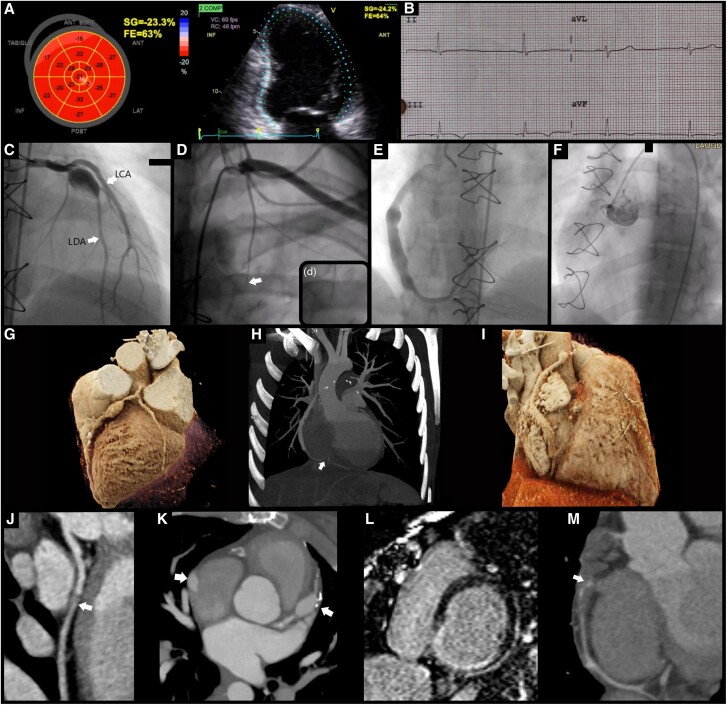
(*A*) A SLGVI of −23.3% and an ejection fraction of 63% by Simpson, along with basal and septal inferior segment akinesia. (*B*) An EKG in sinus rhythm at 48 bpm was recorded, with a P-wave at 60°, QRS at 60°, and T-wave at 0°. Inversion was noted in DIII, aVF, and V1. The PR interval measured 160 ms, V1 exhibited rS, and V6 showed Rs. The QT interval was 490 ms, and the QTc (Fridericia) was 452 ms. (*C*) Catheterization revealed an aneurysm in the middle segment of the anterior descending artery (DA), which had a length of 18 mm and a diameter of 10 mm classified as giant, TIMI 3. Next to the aneurysm site 50–69% stenosis with preserved circumflex artery (CA) TIMI 3. (*D*,d) Catheterization depicted the bypass permeable to the left internal mammary artery (black arrow), which did not continue due to competitive flow from the DA. (*E*) Contrast-enhanced catheterization showed an ectasic hemoduct, TIMI 2 in the right coronary artery, with a diameter of 2.6 mm in its proximal portion and 7.8 mm in its middle and distal portions. (*F*) Thrombosed right coronary artery (TIMI 0) was observed. (*G*) A 3D volumetric reconstruction (VRT) revealed the DA artery aneurysm before stenosis (*C*). (H) Axial maximum intensity projections (MIP) that shows the hemoduct and thrombosis where the right aneurysm was known to be (white arrow). (I) A 3D VRT of the ectasic hemoduct in the right coronary (*E*). (*J*) Angiotomography of the circumflex artery (*C*). (*K*) Angiotomography, DA artery aneurysm (right arrow, *C*) and the hemoduct (left arrow). (*L*) RM inversion recovery sequence in the short-axis basal third revealed transmural infarction in the inferoseptal and inferior lateral regions. (*M*) Confirmation of thrombosis in the right coronary artery.

The importance of follow-up in patients with giant aneurysms remains crucial, as nearly asymptomatic cases still require monitoring for disease progression, especially those involving the LAD and RCA (McCrindle *et al*., 2020). In this patient’s case, anticoagulants and dual antiplatelet therapy were used due to prior CABG. However, aspirin was discontinued due to frequent bleeding episodes. Treatment regimen includes rivaroxaban, clopidogrel, sotalol, and atorvastatin. She was discharged in stable condition and has returned to her daily activities.

## Supplementary Material

qyae116_Supplementary_Data

## Supplementary data

**Funding:** The authors have no funding sources to declare.

**Data availability:** Patient data are not publicly available but are available from the corresponding author on reasonable request. Requests will be subjected to institutional review.

## Lead author biography

Jacobo Sebastian Vera Chavez is a social service medical intern at the National Institute of Cardiology, having graduated from Westhill University in Mexico. With a background in cardiovascular care, he is currently in the process of entering the general surgery specialty, aiming to deepen his expertise in surgical procedures and comprehensive patient care.

